# Comparative Effects of *L. plantarum* CGMCC 1258 and *L. reuteri* LR1 on Growth Performance, Antioxidant Function, and Intestinal Immunity in Weaned Pigs

**DOI:** 10.3389/fvets.2021.728849

**Published:** 2021-11-11

**Authors:** Qingsong Tang, Hongbo Yi, Weibin Hong, Qiwen Wu, Xuefen Yang, Shenglan Hu, Yunxia Xiong, Li Wang, Zongyong Jiang

**Affiliations:** ^1^State Key Laboratory of Livestock and Poultry Breeding, Ministry of Agriculture Key Laboratory of Animal Nutrition and Feed Science in South China, Guangdong Key Laboratory of Animal Breeding and Nutrition, Maoming Branch, Guangdong Laboratory for Lingnan Modern Agriculture, Institute of Animal Science, Guangdong Academy of Agricultural Sciences, Guangzhou, China; ^2^College of Animal Science, Institute of Animal Nutrition and Feed Science, Guizhou University, Guiyang, China

**Keywords:** *Lactobacillus plantarum*, *Lactobacillus reuteri*, antioxidant function, intestinal immunity, weaned pigs

## Abstract

*Lactobacillus plantarum* CGMCC 1258 and *Lactobacillus reuteri* LR1 are two important strains of probiotics. However, their different advantages in the probiotic effect of weaned pigs are still poorly understood. Therefore, the study was to investigate the comparative effects of dietary supplementation of *L. plantarum* CGMCC 1258 and *L. reuteri* LR1 on growth performance, antioxidant function, and intestinal immunity in weaned pigs. Ninety barrows [initial body weight (BW) = 6.10 ± 0.1 kg] 21 days old were randomly divided into 3 treatments with 5 replicates, each replicate containing 6 pigs. Pigs in control (CON) were fed a basal diet, and the basal diets supplemented with 5 × 10^10^ CFU/kg *L. plantarum* CGMCC 1258 (LP) or *L. reuteri* LR1 (LR) for 42 days, respectively. The results showed that LP increased (*p* < 0.05) serum superoxide dismutase (SOD), and decreased (*p* < 0.05) serum malondialdehyde (MDA) and the expression and secretion of interleukin-1β (IL-1β), tumor necrosis factor-α (TNF-α), and interferon-γ (IFN-γ) in intestinal mucosa, but has no significant effect on growth performance and diarrheal incidence. However, LR increased (*p* < 0.05) final BW and average daily gain (ADG), reduced (*p* < 0.05) 29–42-day diarrheal incidence, decreased (*p* < 0.05) the expression and secretion of IL-1β, IL-6, TNF-α, and IFN-γ, and increased (*p* < 0.05) the expression of transforming growth factor-β (TGF-β) in intestinal mucosa. In addition, the serum glutathione peroxidase (GSH-PX), mRNA relative expression of Na+-K+-2Cl– co-transporter 1 (NKCC1) and cystic fibrosis transmembrane conductance regulator (CFTR) and the content of toll-like relative (TLR2) and TLR4 in the jejunum, and secretory immunoglobulin (sIgA) content of ileal mucosa were higher (*p* < 0.05) than LP. Collectively, dietary *L. plantarum* CGMCC 1258 improved intestinal morphology, intestinal permeability, intestinal immunity, and antioxidant function in weaned pigs. Dietary *L. reuteri* LR1 showed better growth performance, a lower incidence of diarrhea, better intestinal morphology, and a higher extent of immune activation in weaned pigs.

## Introduction

Early weaning is often associated with a range of disorders in pigs including digestive upset, low feed intake, poor immunocompetence, diarrhea, and reduced growth performance (1, 2). After the widespread restriction of the use of growth-promoting antibiotics, probiotic additives have played an important role in improving immune response, intestinal microbial balance, and the pH of the gastrointestinal tract of weaned pigs (3). *Lactobacillus* is a widely used probiotic agent. *Lactobacillus plantarum* and *Lactobacillus reuteri* have been used in vertebrates such as pigs, chickens, and humans (4). *L. plantarum* and *L. reuteri* improve intestinal health by producing exopolysaccharides to increase intestinal adhesion and colonization of probiotics. Currently, *L. plantarum* and *L. reuteri* may promote host immunity and intestinal physiological functions by coregulating pro-inflammatory and anti-inflammatory cytokines that has been proven in many ways (5, 6). In addition, previous studies have shown that *L. plantarum* CJLP243 (1 × 10^10^ CFU/kg) or *L. plantarum* CGMCC 1258 (5 × 10^10^ CFU/kg) can improve growth performance and enhance the defense of intestinal epithelial barrier in weaned pigs challenged by *Escherichia coli* (7, 8). *L. plantarum* ZJ316 also improved the growth performance of weaned pigs under normal feeding conditions (9). For *L. reuteri, L. reuteri* D8 promotes the development of intestine mucosal system and maintains intestinal mucosal barrier (10). Previous studies in this laboratory have showed that a strain of *L. reuteri* LR1 isolated from the feces of healthy piglets showed bile resistance and notable acid (11). Dietary *L. reuteri* LR1 supplemented at 5 × 10^10^ CFU/kg improved growth performance, epithelial barrier function, and enhanced amino acid metabolism in weaned pigs (12, 13). However, under the premise that the two strains of *L. plantarum* CGMCC 1258 and *L. reuteri* LR1 are known to have good probiotic effects on weaned pigs, their different advantages in the probiotic effect on weaned pigs are still lacking. Hence, the present study was conducted to investigate the differential effects of *L. plantarum* CGMCC 1258 and *L. reuteri* LR1 on the growth performance, antioxidant function, and intestinal immunity in weaned pigs.

## Materials and Methods

These experiments were conducted in accordance with Chinese guidelines for animal welfare and experimental protocols, and all animal procedures were approved by the Animal Care and Use Committee of Guangdong Academy of Agricultural Sciences (Permit Number: GAASIAS-2015-012). The *L. plantarum* CGMCC 1258 strain was provided by Dr. Hang Xiaomin (Institute of Science Life of Onlly, Shanghai Jiao Tong University, Shanghai, China), and the strain was originally isolated from the feces of healthy infants (14). The *L. reuteri* LR1 strain was originally isolated from the feces of healthy 35-day-old weaned pigs in our laboratory (11).

### Animals and Diets

A total of 90 barrows [Duroc × (Landrace × Yorkshire), 21 d of age, body weight (BW) = 6.10 ± 0.1 kg] were randomly allocated to three groups (five replicates per group and six pigs per replicate). Control group (CON) were fed a corn–soybean meal basal diet, *L. plantarum* CGMCC 1258 group (LP) were fed the basal diet supplemented with 5 × 10^10^ CFU/kg *L. plantarum* CGMCC 1258, and *L. reuteri* LR1 group (LR) were fed the basal diet supplemented with 5 × 10^10^ CFU/kg *L. reuteri* LR1 for 42 days. Experimental diets were formulated to meet the nutrient requirements for pigs proposed by the National Research Council (15), and the ingredient compositions and nutrient levels of the basal diets are listed in Table 1. The measurements of crude protein (CP), Ca, and P refer to GB/T 6432-2018 (China), GB/T 6436-2018 (China), and GB/T 6437-2018 (China), respectively. The diets used in the experiment were all mash feed. The pigs were provided feed and water *ad libitum* throughout the experiment.

**Table 1:** The formulations and chemical composition of the basal diet (as-fed basis).

**Ingredient**	**%**
Corn	57.55
Soybean meal	27.59
Whey powder	5.00
Soybean oil	1.89
Fish meal	3.00
*L*-Lys·HCl	0.73
*L*-Thr	0.29
*DL*-Met	0.24
*L*-Trp	0.03
CaHPO_4_	1.40
Limestone	0.85
Wheat middlings	0.30
NaCl	0.14
Premix^a^	1.00
Total	100.00
**Energy and nutrient composition**	
NE, MJ/kg	10.51
CP, %	20.01
Standardized ileal digestible Lys, %	1.57
Standardized ileal digestible Met + Cys, %	0.82
Standardized ileal digestible Thr, %	0.94
Standardized ileal digestible Trp, %	0.25
Ca, %	0.85
Total P, %	0.70
Available phosphorus P, %	0.48

a *The premix provided following per kg of the diet: vitamin A, 5,500 IU; vitamin D, 500 IU; vitamin E, 66.1 IU; vitamin B_12_ 28.2 μg; vitamin B_2_, 5.1 mg; pantothenic acid, 12.6 mg; niacin, 29.8 mg; choline chloride, 540 mg; Mn (MnSO_4_·H_2_O), 100 mg; Zn (ZnSO_4_·H_2_O), 100 mg; Fe (FeSO_4_·H_2_O), 100 mg; Cu (CuSO_4_·5H_2_O), 150 mg; Co (CoSO_4_·7H_2_O) 1 mg; and Se (Na_2_SeO_3_), 0.48 mg*.

*Except for the calculated values of net energy, available phosphorus, and standardized ileal digestible AA, dietary nutrients were all measured values*.

### Sample Collection

For each pen, two pigs were randomly selected for blood collection and slaughter sampling. Blood samples and tissue sampling from all weaned pigs were completed on day 43. Blood samples were collected intravenously into 10-ml vacuum tubes without anticoagulant, centrifuged at 3,000 × g at 4°C for 15 min to obtain serum, and stored at −80°C until further assay. After blood collection, one pig per pen was anesthetized by intravenous injection of pentobarbital sodium (30 mg/kg BW) and killed by bloodletting. Approximately 1-cm lengths of middle duodenum, middle jejunum, and distal ileum specimens were collected without rinsing and fixed in 4% paraformaldehyde. Approximately 10-cm lengths of jejunum and ileum were cut open to expose the intestinal lumen, rinsed with phosphate buffered saline, and the mucosa were scraped by sterile glass microscope slide, and then the samples were quickly frozen in liquid nitrogen and stored at −80°C until analyses.

### Performance and Diarrhea Measurements

Feed intake was measured every day during the entire experiment, and pigs were weighed on days 0 and 42 to calculate average daily gain (ADG), average daily feed intake (ADFI), and gain:feed ratio (G/F). In addition, the diarrhea was observed and recorded in each pen at 9:00 and 16:00 every day. Diarrhea is evaluated according to the shape of the stool; strips or pellets are normal stools, while flat or liquid stools are diarrhea stools. Diarrhea incidence was calculated at the end of the experiment for each enclosure from 1 to 14 days, 15 to 28 days, 28 to 42 days, and 1 to 42 days. Diarrhea incidence was calculated according to the formula: diarrheal incidence (%) = [total number of pigs with diarrhea in each pen × diarrhea days/(6 pigs × the number of days)] × 100.

### Analysis of Intestinal Morphology

The collected fixed samples ileum, jejunum, and duodenum were dehydrated and embedded in paraffin. Sections of 5 μm thickness were stained coated with H&E. Nine well-oriented and intact villi and adjacent crypts were measured each section using Image-Pro software (Media Cybernetics, Rockville, MD), and the villus height to crypt depth ratio (V/C) was calculated. The images were obtained by an Axio Scope A1 microscope (Zeiss, Germany).

### Analysis of Antioxidant Function and Intestinal Cytokines

Lactate dehydrogenase (LDH, A020-2-2), the activities of superoxide dismutase (SOD, A001-3-2), malondialdehyde (MDA, A003-1-2), glutathione peroxidase (GSH-PX, A005-1-2), urea nitrogen (SUN, C013-2-1), and glucose (GLU, F006-1-1) in serum were estimated using a commercial kit (Nanjing Jiancheng Bioengineering Institute, Nanjing, China). The contents of immunoglobulin G (IgG, FU-Z076), lipopolysaccharide (LPS, YS04547B), insulin-like growth factor 1 (IGF-1, FU-Z135), and diamine oxidase (DAO, FU-Z050) in serum were estimated using ELISA kits (Beijing FangCheng Bioengineering Institute, Beijing, China). To obtain a 10% intestinal mucosa supernatant, 0.4 g of intestinal mucosa was added to 3.6 ml of 0.86% normal saline, homogenized in ice water with a tissue homogenizer, and centrifuged at 3,000 × g at 4°C for 10 min. The content of total protein in the supernatant was determined by BCA protein analysis kit (Thermo Fisher Scientific, Waltham, MA, 23227). The levels of interleukin-1β (IL-1β, H002), IL-6, tumor necrosis factor-α (TNF-α, FU-Z149), transforming growth factor-β (TGF-β, FU- FU-Z014), interferon-γ (IFN-γ, FU-Z054), secretory immunoglobulin (sIgA, H108-2) (Beijing FangCheng Bioengineering Institute), and TLR2 (ml027585) and TLR4 (ml027583) in intestinal mucosa were estimated using ELISA kits (Mlbio Bioengineering, Shanghai, China).

### Real-Time PCR for Relative Measurement of Intestinal Diarrhea-Related Ion Channel Genes and Cytokines

Total RNA of the mucosa of jejunum and ileum was extracted following the Trizol Reagent Instructions (Invitrogen, Carlsbad, CA). The total RNA was quantified using a NanoDrop 1000 spectrophotometer (Thermo Fisher Scientific, Waltham, MA). RNA purity was assessed by determining the ratio of absorbance at 260 nm to that at 280 nm, and RNA (2 μg) was used to generate cDNA in a volume of 20 μl using a PrimeScript II 1st Strand cDNA Synthesis Kit (Takara, Tokyo, Japan). PCR amplification was performed in a total volume of 20 μl containing 10 μl of master mix (SYBR PCR Master Mix; Applied Biosystems), 1.0 μl of gene-specific primers (Table 2), 6.0 μl of RNAse-free water, and 2 μl 10-fold diluted cDNA. The thermocycler protocol consisted of 1 min at 95°C followed by 39 cycles of 10 s at 95°C, 30 s at 60°C, and 30 s at 72°C. β-Actin was used as a housekeeping gene. The fold changes were calculated for each sample using the 2^−ΔΔCt^ method, and data for each target transcript were normalized to control pigs; ΔΔC_T_ = (C_T,Target_ – C_T,β−actin_)_Treatment_ – (C_T,Target_ – C_T,β−actin_)_Control_.

**Table 2:** Primers for the real-time PCR analysis.

**Gene**	**Accession number**	**Sequence (5′-3′)**	**Size (bp)**
NKCC1	CU855646.2	Forward: CAAGAAAAGGTGCTGTGTC	109
		Reverse: GTAAGGACGCTCTGATGATT	
CFTR	AY585334.1	Forward: TTCCTCGTAGTCCTCGCC	204
		Reverse: GGTCAGTTTCAGTTCCGTTTG	
IL-1β	NM214055.1	Forward: CTCCAGCCAGTCTTCATTGTTC	132
		Reverse: TGCCTGATGCTCTTGTTCCA	
IL-6	M80258.1	Forward: TACATCCTCGGCAAAATC	168
		Reverse: TCTCATCAAGCAGGTCTCC	
IFN-γ	JF906510	Forward: TGTTTTTCTGGCTCTTACTGC	99
		Reverse: CCTTTGAATGGCCTGGTT	
TGF-β	NM_214379.1	Forward: GAAGCGCATCGAGGCCATTC	162
		Reverse: GGCTCCGGTTCGACACTTTC	
TNF-α	NM_214022.1	Forward: CCAATGGCAGAGTGGGTATG	116
		Reverse: TGAAGAGGACCTGGGAGTAG	
TLR2	NM_213761	Forward: ACGGACTGTGGTGCATGAAG	101
		Reverse: GGACACGAAAGCGTCATAGC	
TLR4	NM_001113039	Forward: CATACAGAGCCGATGGTG	136
		Reverse: CCTGCTGAGAAGGCGATA	
β-Actin	DQ845171	Forward: CGGGACATCAAGGAGAAGC	273
		Reverse: ACAGCACCGTGTTGGCGTAGAG	

*NKCC1, Na+-K+-2Cl– co-transporter 1; CFTR, cystic fibrosis transmembrane conductance regulator; IL, interleukin; IFN-γ, interferon-γ; TGF-β, transforming growth factor-β; TLR, toll-like receptor*.

### Statistical Analyses

The pen was the experimental unit. Statistical significance analysis was determined by one-way ANOVA with Tukey's test using SPSS 19.0 software (SPSS Inc., Chicago, IL). All data were expressed as the means ± SEM. The differences were significant at *p* < 0.05.

## Results

### Effects of *L. plantarum* CGMCC 1258 and *L. reuteri* LR1 on Growth Performance and Diarrhea in Weaned Pigs

LR but not LP increased final BW (*p* = 0.013) and ADG (*p* = 0.013), and reduced (*p* = 0.025) 29–42-day diarrheal incidence compared with CON (Table 3). However, no significant differences were observed on ADFI and neither on 1–42-day diarrheal incidence between treatments (*p* = 0.154).

**Table 3:** Effects of *L. plantarum* CGMCC 1258 and *L. reuteri* LR1 on growth performance and diarrhea of weaned pigs.

**Item**	**Treatments**			**SEM**	***P*-value**
	**CON**	**LP**	**LR**		
Initial BW, kg	6.10	6.10	6.11	0.065	0.893
Final BW, kg	14.9^b^	16.4^a,b^	17.5^a^	0.43	0.017
ADFI, g/day	321	358	384	13.4	0.154
ADG, g/day	207^b^	244^a,b^	274^a^	10.3	0.017
G/F	0.664	0.681	0.714	0.0219	0.686
**Diarrhea incidence, %**					
1–14 days	23.9	22.0	18.5	1.86	0.509
15–28 days	22.1	21.2	18.8	2.11	0.829
29–42 days	21.5^a^	16.5^a,b^	13.2^b^	1.72	0.046
1–42 days	21.4	19.0	16.0	1.42	0.309

*n = 5*.

*CON, a basal diet; LP, a basal diet supplemented with 5 × 10^10^ CFU/kg L. plantarum CGMCC 1258; LR, a basal diet supplemented with 5 × 10^10^ CFU/kg L. reuteri LR1*.

a,b *Values within a row with different superscripts differ significantly at p < 0.05*.

### Effects of *L. plantarum* CGMCC 1258 and *L. reuteri* LR1 on Antioxidant Function in Weaned Pigs

LR increased serum GLU (*p* = 0.007) compared with CON and LP (Table 4). In addition, the LP increased (*p* = 0.043) serum SOD compared with CON. LR increased serum GSH-Px compared with both CON (*p* = 0.002) and LP (*p* = 0.001).

**Table 4:** Effects of *L. plantarum* CGMCC 1258 and *L. reuteri* LR1 on serum indices of pigs.

**Item**	**Treatments**			**SEM**	***P*-value**
	**CON**	**LP**	**LR**		
GLU (mmol/L)	7.79^b^	7.87^b^	8.88^a^	0.146	0.001
SUN (mmol/L)	20.6	18.2	18.1	0.74	0.093
IGF-1 (μg/L)	80.3	79.0	80.4	4.61	0.991
IgG (μg/ml/L)	156	156	175	6.0	0.355
SOD (U/ml)	93.0^b^	102^a^	98.0^a,b^	1.41	0.052
GSH-Px (U/ml)	417^b^	406^b^	505^a^	12.2	<0.001
LDH (U/ml)	2,494	2,420	2,403	43.4	0.673
MDA (nmol/ml)	2.31	1.63	2.01	0.144	0.153

*n = 5*.

*CON, a basal diet; LP, a basal diet supplemented with 5 × 10^10^ CFU/kg L. plantarum CGMCC 1258; LR, a basal diet supplemented with 5 × 10^10^ CFU/kg L. reuteri LR1*.

a,b *Values within a row with different superscripts differ significantly at p < 0.05*.

### Effects of *L. plantarum* CGMCC 1258 and *L. reuteri* LR1 on Intestinal Morphology in Weaned Pigs

LP increased duodenal villus height compared with CON (*p* = 0.0003) and LR (*p* = 0.018), and the jejunal villus height of LR was higher (*p* = 0.041) than that of CON (Table 5). In addition, LP increased (*p* = 0.001) the V/C of duodenum compared with CON. LR increased the V/C of jejunum (*p* = 0.011) and ileum (*p* = 0.018) compared with CON.

**Table 5:** Effects of *L. plantarum* CGMCC 1258 and *L. reuteri* LR1 on intestinal morphology in weaned pigs.

**Item**	**Treatments**			**SEM**	***P*-value**
	**CON**	**LP**	**LR**		
**Duodenum**					
Villus height, μm	351^b^	505^a^	415^b^	19.3	<0.001
Crypt depth, μm	435	307	347	37.8	0.106
Villus height/crypt depth	0.850^b^	1.72^a^	1.27^a,b^	0.1163	0.001
**Jejunum**					
Villus height, μm	387^b^	415^a,b^	480^a^	22.7	0.045
Crypt depth, μm	314	273	221	27.6	0.113
Villus height/crypt	1.28^b^	1.60^a,b^	2.24^a^	0.180	0.013
depth					
**Ileum**					
Villus height, μm	359	408	425	31.2	0.331
Crypt depth, μm	261	247	173	30.5	0.137
Villus height/crypt depth	1.41^b^	1.96^a,b^	2.42^a^	0.223	0.023

*n = 5*.

*CON, a basal diet; LP, a basal diet supplemented with 5 × 10^10^ CFU/kg L. plantarum CGMCC 1258; LR, a basal diet supplemented with 5 × 10^10^ CFU/kg L. reuteri LR1*.

a,b *Values within a row with different superscripts differ significantly at p < 0.05*.

### Effects of *L. plantarum* CGMCC 1258 and *L. reuteri* LR1 on Intestinal Permeability in Weaned Pigs

**Figure 1:**
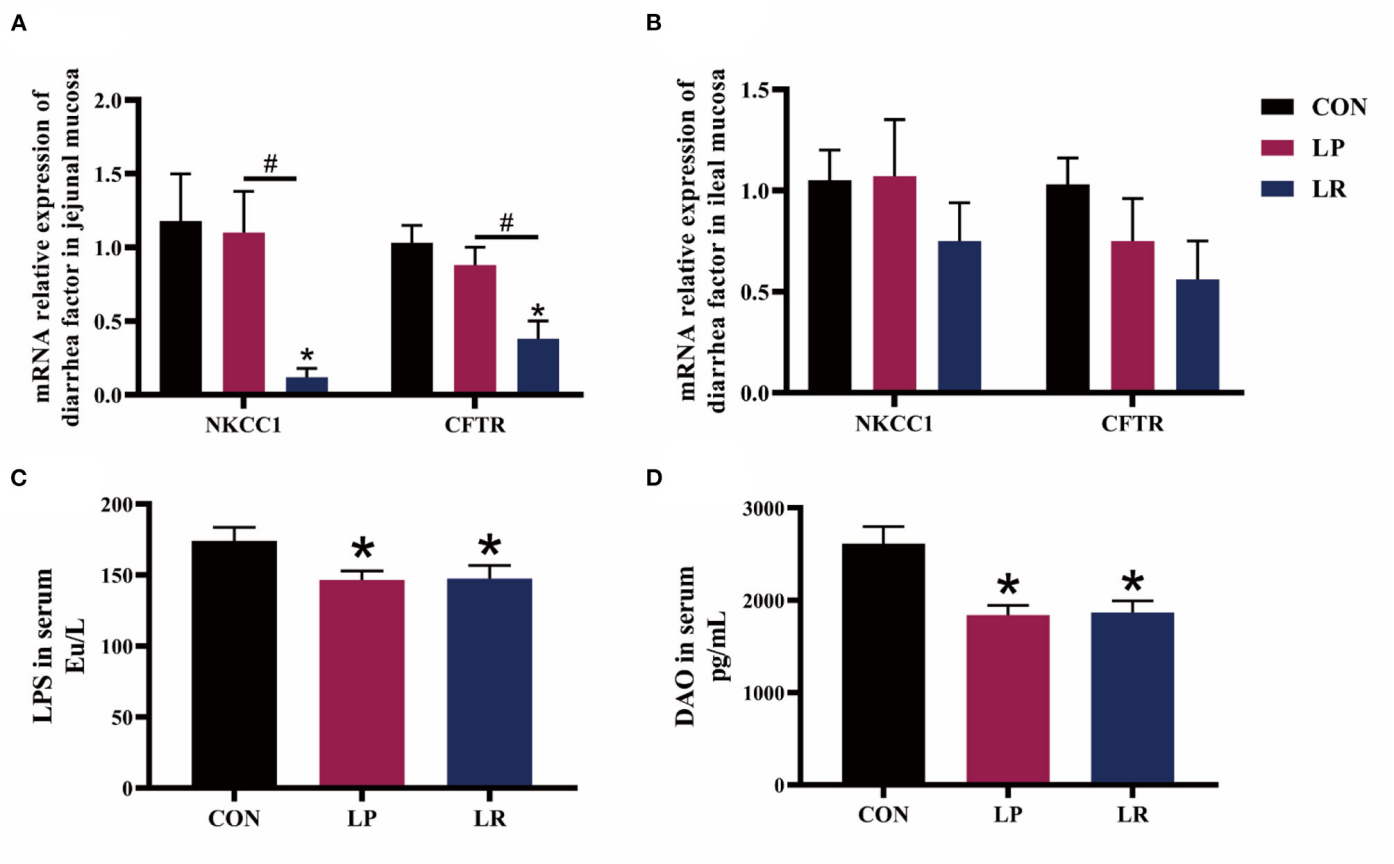
Effects of *L. plantarum* CGMCC 1258 and *L. reuteri* LR1 on diarrhea-related ion channel genes and intestinal permeability in weaned pigs. The relative mRNA expression levels of NKCC1 and CFTR in jejunal mucosa **(A)** and ileal mucosa **(B)** were determined via real-time PCR. Levels of LPS **(C)** and DAO **(D)** in serum determined by ELISA. NKCC1, Na+-K+-2Cl– co-transporter 1; CFTR, cystic fibrosis transmembrane conductance regulator; LPS, lipopolysaccharide; DAO, diamine oxidase. All data are expressed as the mean ± SEM (*n* = 5). Differences were determined by one-way ANOVA followed by Tukey test. *p < 0.05 compared with CON, ^#^*p* < 0.05 compared with LP.

The expression of Na+-K+-2Cl– co-transporter 1 (NKCC1) in jejunal mucosa was decreased by LR compared with CON (*p* = 0.025) and LP (*p* = 0.029) (Figure 1). Meanwhile, LR decreased the expression of and cystic fibrosis transmembrane conductance regulator (CFTR) in jejunal mucosa compared with CON (*p* = 0.036) and LP (*p* = 0.038). In addition, both LP (*p* = 0.001) and LR (*p* = 0.002) decreased serum DAO above CON, similar to the effect of LP (*p* = 0.0002) and LR (*p* = 0.0004) on serum LPS compared with CON.

### Effects of *L. plantarum* CGMCC 1258 and *L. reuteri* LR1 on Intestinal Cytokines in Weaned Pigs

**Figure 2:**
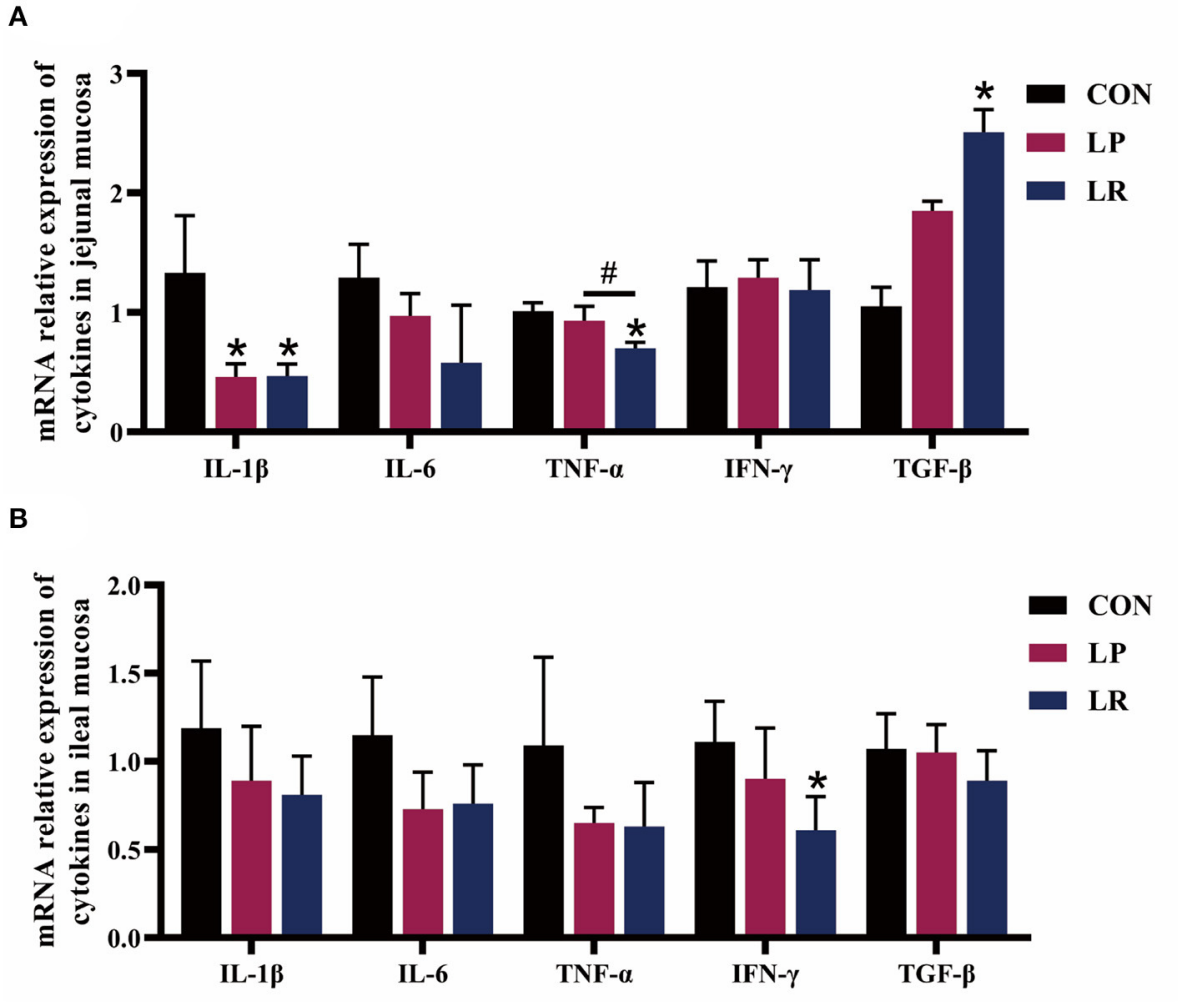
Effect of *L. plantarum* CGMCC 1258 and *L. reuteri* LR1 on the expression of cytokines in intestinal in weaned pigs. The relative mRNA expression levels of IL-1β, IL-6, TNF-α, IFN-γ, and TGF-β in the jejunal mucosa **(A)** and ileal mucosa **(B)** were determined via real-time PCR. All data are expressed as the mean ± SEM (*n* = 5). Differences were determined by one-way ANOVA followed by Tukey test. *p < 0.05 compared with CON, ^#^*p* < 0.05 compared with LP.

Figure 2, Table 6 show that both LP (*p* = 0.029) and LR (*p* = 0.025) decreased IL-1β transcripts in jejunal mucosa and LP decreased (*p* = 0.004) ileal mucosa content of IL-1β compared with CON. LR decreased jejunal mucosa (*p* = 0.041) and ileal mucosa (*p* = 0.004) content of IL-6 compared with CON. The relative mRNA expression of TNF-α in jejunal mucosa was decreased by LR compared with CON (*p* = 0.046) and LP (*p* = 0.049). Both LP (*p* = 0.017) and LR (*p* = 0.011) decreased the TNF-α content of jejunal mucosa compared with CON. LR decreased (*p* = 0.039) the mRNA expression of IFN-γ in ileum mucosa compared with CON. LR increased (*p* < 0.05) the mRNA expression of TGF-β in jejunal mucosa compared with CON, and the TGF-β content in jejunal mucosa was increased in pigs fed LR compared with CON (*p* = 0.001) and LP (*p* = 0.043). In addition, LP (*p* = 0.0002) and LR (*p* = 0.0003) increased the sIgA content of the jejunal mucosa compared with CON, and concentrations of sIgA in ileal mucosa in LR was higher than in CON (*p* = 0.018) and LP (*p* = 0.009).

**Table 6:** Effects of *L. plantarum* CGMCC 1258 and *L. reuteri* LR1 on intestinal mucosal cytokines and sIgA concentrations of weaned pigs.

**Item**	**Treatments**			**SEM**	***P*-value**
	**CON**	**LP**	**LR**		
**Jejunal mucosa**					
IL-1β, pg/ml	148	141	130	6.5	0.571
IL-6, pg/ml	20.5^a^	18.1^a,b^	16.5^b^	0.68	0.049
TNF-α, pg/ml	109^a^	88.8^b^	90.3^b^	3.44	0.007
IFN-γ, pg/ml	314	240	246	14.9	0.066
TGF-β, pg/ml	22.5^b^	27.9^b^	33.8^a^	1.48	0.001
sIgA, μg/ml	65.9^b^	75.1^a^	77.6^a^	1.50	<0.001
**Ileal mucosa**					
IL-1β, pg/ml	103^a^	64.9^b^	101^a^	5.83	0.003
IL-6, pg/ml	19.3^a^	16.4^a,b^	14.8^b^	0.66	0.005
TNF-α, pg/ml	63.6	48.4	57.6	3.74	0.260
IFN-γ, pg/ml	207	193	200	9.1	0.841
TGF-β, pg/ml	26.2	26.4	27.6	1.27	0.901
sIgA, μg/ml	24.9^b^	23.5^b^	35.7^a^	2.85	0.006

*n = 5*.

*CON, a basal diet; LP, a basal diet supplemented with 5 × 10^10^ CFU/kg L. plantarum CGMCC 1258; LR, a basal diet supplemented with 5 × 10^10^ CFU/kg L. reuteri LR1*.

a,b *Values within a row with different superscripts differ significantly at p < 0.05*.

### Effects of *L. plantarum* CGMCC 1258 and *L. reuteri* LR1 on TLRs in the Intestinal Mucosa in Weaned Pigs

**Figure 3:**
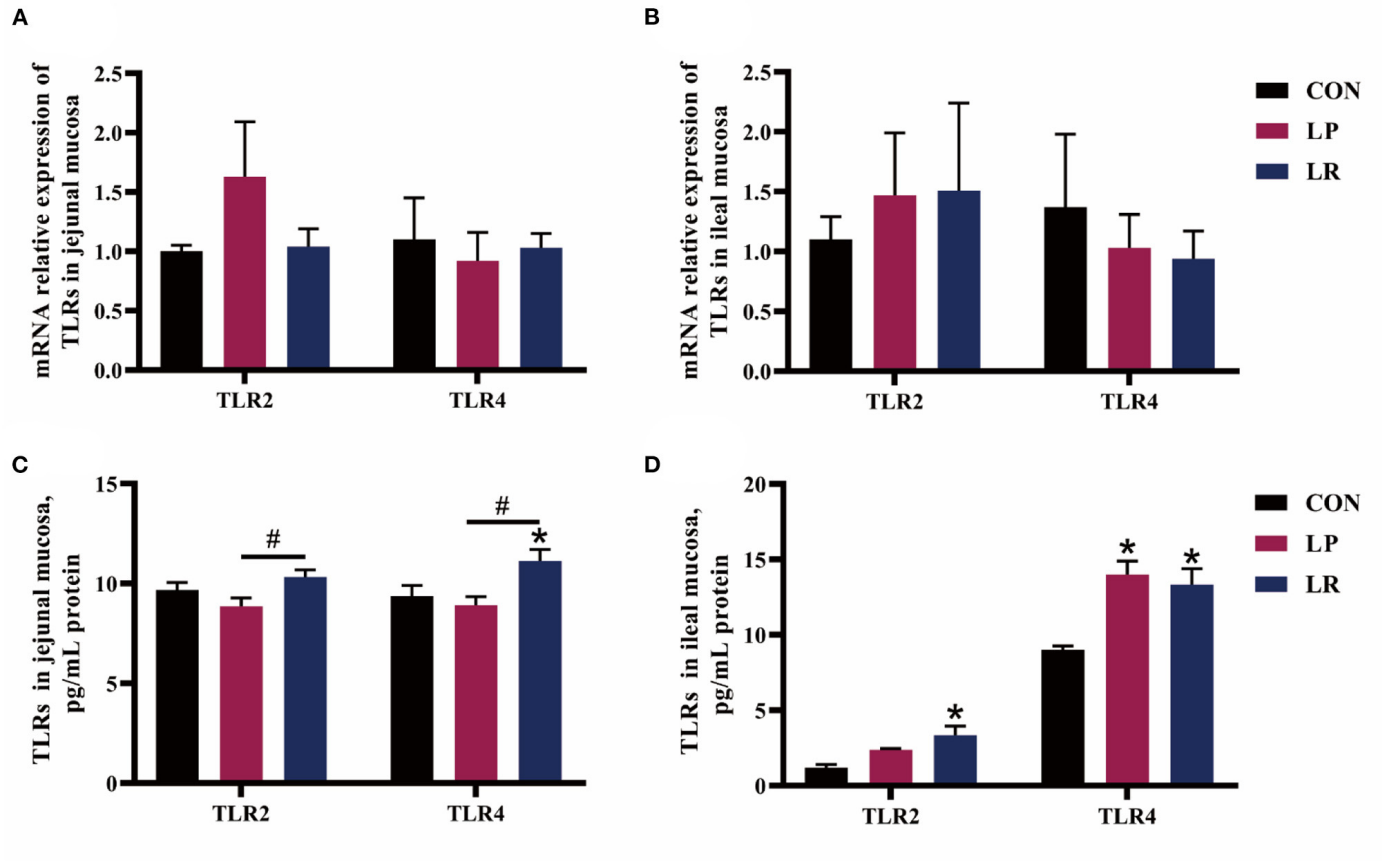
Effects of *L. plantarum* CGMCC 1258 and *L. reuteri* LR1 on TLRs in intestinal mucosa in weaned pigs. The relative mRNA expression levels of TLRs in the jejunal mucosa **(A)** and ileal mucosa **(B)** were determined via real-time PCR. Levels of TLRs in the jejunal mucosa **(C)** and ileal mucosa **(D)** determined by ELISA. All data are expressed as the mean ± SEM (*n* = 5). Differences were determined by one-way ANOVA followed by Tukey test. ^*^p < 0.05 compared with CON, ^#^*p* < 0.05 compared with LP.

LR increased (*p* = 0.003) content of TLR2 in the ileal mucosa compared with CON, and the content of TLR2 in the jejunal mucosa of LR is higher (*p* = 0.015) than that of LP (Figure 3). Both LP (*p* = 0.001) and LR (*p* = 0.003) increased content of TLR4 in ileal mucosa compared with CON, and LR increased content of TLR4 in jejunal mucosa compared with CON (*p* = 0.012) and LP (*p* = 0.003).

## Discussion

In the present study, we compared the effects of *L. plantarum* CGMCC 1258 and *L. reuteri* LR1 on the growth performance, antioxidant function, and intestinal immune function of weaned pigs. The results showed that both dietary *L. plantarum* CGMCC 1258 and *L. reuteri* LR1 supplementation at 5 × 10^10^ CFU/kg improved the antioxidant function, intestinal morphology, and intestinal immunity of weaned pigs, and they are consistent in improving intestinal permeability. However, *L. plantarum* CGMCC 1258 has a better effect on SOD and *L. reuteri* LR1 has better effects on ADG, diarrheal incidence, GLU, GSH-Px, intestinal morphology, and intestinal immunity.

Generally, the level of GLU is positively correlated with the digestion and absorption of carbohydrates in the intestine of pigs. In this study, *L. reuteri* LR1 but not *L. plantarum* CGMCC 1258 significantly increased the GLU content in the serum. The results suggest that *L. reuteri* LR1 improves serum GLU of weaned pigs, which was more advantageous than *L. plantarum* CGMCC 1258.

Weaning often induces a large number of reactive oxygen radicals in the piglets, causing oxidative stress and resulting in reduced piglet production performance and immune function (16). SOD and GSH-Px can scavenge reactive oxygen radicals in the body and are the main antioxidant enzymes in the body. MDA is a small molecule product produced at the termination stage of lipid peroxidation reaction, and its content can reflect the degree of lipid oxidation caused by reactive oxygen species in the organism (17). The strain of *L. plantarum* 423, *L. plantarum* 200655, and *L. plantarum* RG14 showed strong free radical–scavenging activity, and exert strong antioxidant capacity of human umbilical vein endothelial cells, human colon adenocarcinoma cell line, and post-weaning lambs, respectively (18–20). According to the mechanisms related to probiotic effects, *L. plantarum* and *L. reuteri* have been reported to limit excessive amounts of reactive radicals against oxidative stress *in vivo* (21, 22). Another study showed that dietary supplementation of *L. plantarum* ZLP001 increased the activity of serum SOD and GSH-Px in weaned pigs and reduced MDA content (23). The feeding with *L. reuteri* KT260178 increased the plasma total antioxidant capacity (T-AOC), SOD, and GSH-Px, which did not increase MDA in suckling piglets (24). However, the antioxidant function of *L. plantarum* CGMCC 1258 and *L. reuteri* LR1 has not been reported. In this study, *L. plantarum* CGMCC1258 increased serum SOD enzyme activity of weaned pigs, while *L. reuteri* LR1 increased the enzyme activity of GSH-Px. Taken together, *L. reuteri* LR1 improved the antioxidant function mainly by regulating GSH-Px, while *L. plantarum* CGMCC 1258 mainly affects SOD.

Probiotics are often used to improve the performance and intestinal health of pigs. Our data showed that the *L. reuteri* LR1 increased the ADG, but *L. plantarum* CGMCC 1258 has no significant effects on the growth performance of weaned pigs. The results of *L. plantarum* CGMCC 1258 in this experiment was different from a previous study, but the results of *L. reuteri* LR1 are consistent. Other strains of *L. plantarum* (such as CJLP243) and the our previous researched on *L. plantarum* CGMCC 1258 strain can improve the growth performance of weaned pigs by *Escherichia coli* challenge (7, 8), and *L. plantarum* ZJ316 and *L. reuteri* LR1 also improved the growth performance of weaned pigs under normal feeding conditions (9, 12, 25). We have also previously study that the *L. reuteri* LR1 strain improved the growth performance of weaned pigs under normal feeding conditions (12). Under normal feeding conditions of this experiment, there was no significant effect of *L. plantarum* CGMCC 1258 on growth performance of weaned pigs, which is quite different from the past, which may be related to the isolation of *L. plantarum* CGMCC 1258 strain from infant feces and the different experimental conditions. However, its influence mechanism needs more in-depth study.

In the present study, *L. reuteri* LR1 but not *L. plantarum* CGMCC1258 reduced the incidence of diarrhea in weaned pigs during the period of 29–42 days. Lee et al. (7), Yang et al. (8), and Suo et al. (9) showed that *L. plantarum* ZJ316 was effective in reducing diarrhea incidence in weaned pigs, but the reductions in diarrhea incidence with *L. plantarum* CGMCC1258 were all in the *Escherichia coli* challenge feeding mode (7–9). Probiotics play a detoxification role by inhibiting the reproduction of pathogenic bacteria, removing intestinal metabolites and bacteriocins (26). In the absence of *E. coli* challenge and under conditions of good intestinal health, *L. plantarum* CGMCC1258 was unable to exert significant antimicrobial and detoxification abilities in the intestine of weaned pigs. This may be the reason why *L. plantarum* CGMCC1258 is different from previous studies. In addition, stimulants such as enterotoxin and inflammatory mediators stimulate intestinal mucosal cells, and activate CFTR at the top of intestinal mucosal cells through G protein-coupled signaling pathways and phosphorylation, leading to a large amount of intracellular Cl– and water secretion, causing watery diarrhea (27). The activities of basolateral transport proteins NKCC1 are the rate-limiting steps of ion and fluid secretions in Cl-secreting epithelia (28, 29). For diarrhea-related ion channel genes, we found that *L. reuteri* LR1 reduced the expression of NKCC1 and CFTR in the intestine of piglets, which may be one of the reasons for its reduction of diarrhea. Taken together, *L. reuteri* LR1 showed better effects on reduced diarrhea of weaned pigs than *L. plantarum* CGMCC 1258.

The intestinal barrier plays an important role in resisting the invasion of intestinal bacteria and pathogenic allergens into the mucosa (30). When the intestinal mucosa is damaged, the increase in intestinal permeability leads to more DAO and LPS from the tissues into the peripheral blood circulation (31, 32). Pan et al. (33) have found that the addition of probiotics (mainly *Bacillus licheniformis* and *Saccharomyces cerevisiae*) could reduce the intestinal damage caused by the enterotoxigenic *Escherichia coli* K88 challenge through reducing the serum DAO content of weaned pigs (33). In this study, both *L. plantarum* CGMCC1258 and *L. reuteri* LR1 reduced the content of DAO and LPS in the serum in weaned pigs. The formation of villi and crypt in the intestine enlarged the surface area of the intestinal mucosa, and not only promoted the efficient absorption of nutrients but also generated a protected stem cell niche (10). We found that *L. plantarum* CGMCC1258 improved the intestinal morphology of the duodenum, while *L. reuteri* LR1 improved the intestinal morphology of the jejunum and ileum. This result showed that *L. plantarum* CGMCC1258 and *L. reuteri* LR1 enhanced the intestinal barrier function, and *L. reuteri* LR1 can better improve the intestinal morphology of weaned pigs.

The increased expression and secretion of pro-inflammatory factors IL-1β, IL-6, TNF-α, and IFN-γ are stimulated by weaning stress or pathogenic invasion (34, 35). The strain of *L. plantarum* CGMCC1258 and *L. plantarum* ACTT 8014 could effectively increase the protein levels of the natural cytotoxic receptor family of natural killer cells, and alleviates the pathological changes of intestinal tissues of animal intestinal inflammation models (36, 37). The *L. plantarum* 299v facilitates the gut health of suckling piglets by improved the intestinal morphology and intestinal barrier function and microflora (38). In this study, *L. plantarum* CGMCC1258 reduced the gene expression of IL-1β and the content of IL-1β and TNF-α in the intestinal mucosa, while *L. reuteri* LR1 reduced the gene expression of IL-1β, TNF-α, and IFN-γ and the content of IL-6 and TNF-α, and increased the TGF-β expression. Yi et al. (12) found that *L. reuteri* LR1 increased the content of TGF-β in the ileum and improve the intestinal immunity of weaned pigs (12). Collectively, these two strains of *Lactobacillus* have great differences in regulating the expression of IL-1β, IL-6, TNF-α, and TGF-β in the intestinal mucosa, and *L. reuteri* LR1 showed better anti-inflammatory ability than *L. plantarum* CGMCC 1258. The different effects of *L. reuteri* LR1 and *L. plantarum* CGMCC 1258 on intestinal immunity in weaned pigs may be related to the different hosts from which the strains originate, with *L. reuteri* LR1 from piglet feces readily attaching to the gastrointestinal tract and acting, while *L. plantarum* CGMCC 1258 from infants has lower effects because the piglet probably has not evolved to specifically recognize this strain.

The sIgA has been proven as the first line of defense in intestinal mucosa, effectively preventing the adhesion and penetration of pathogen in intestinal epithelial cells (39). The TLRs play an important role in recognizing bacterial signals and initiating intestinal immune responses. The TLR2 detects lipoprotein and peptidoglycans of gram-positive bacteria and gram-negative bacteria, and TLR4 can recognize LPS of gram-negative bacteria (40). The activation of TLR2 enhances the expression of antimicrobial peptides and tight junction proteins (41, 42). The *L. plantarum* 299v or *L. plantarum* CGMCC 1258 increased the expression of tight junction proteins, which was related to the expression of TLR2 in the pig intestine (8, 38). Another study showed that *L. reuteri* LR1 increased the expression of intestinal antimicrobial peptides, tight junction protein, and sIgA secretion, which was related to the increase of TLR2 and TLR4 expression (12). In the present study, *L. plantarum* CGMCC 1258 increased TLR4 levels only in ileum, and *L. reuteri* LR1 increased TLR2 and TLR4 levels in jejunum and ileum. The probiotic preparations containing *L. plantarum* CGMCC 1258 can significantly increase the sIgA content of the ileal mucosa of pigs 21 days after weaning, and also increased the sIgA content of jejunal mucosa (43). We found that dietary supplement *L. plantarum* CGMCC 1258 and *L. reuteri* LR1 increased the sIgA content in the jejunal mucosa of pigs, and *L. reuteri* LR1 can increase the sIgA content in the ileal mucosa. Collectively, these data suggest that *L. plantarum* CGMCC 1258 and *L. reuteri* LR1 may mediate TLRs-related pathways to regulate the secretion of sIgA and improved the intestinal immune response, but LR has a stronger influence.

## Conclusions

In conclusion, dietary *L. plantarum* CGMCC 1258 supplementation at 5 × 10^10^ CFU/kg improved intestinal morphology, intestinal permeability, intestinal immunity, and antioxidant function in weaned pigs. However, dietary *L. reuteri* LR1 supplementation at 5 × 10^10^ CFU/kg showed higher improvements in growth performance, incidence of diarrhea, intestinal morphology, and a higher extent of immune activation in weaned pigs.

## Data Availability Statement

The raw data supporting the conclusions of this article will be made available by the authors, without undue reservation.

## Ethics Statement

These experiments were conducted in accordance with Chinese guidelines for animal welfare and experimental protocols, and all animal procedures were approved by the Animal Care and Use Committee of Guangdong Academy of Agricultural Sciences (Permit Number: GAASIAS-2015-012).

## Author Contributions

QT, HY, and LW: conceptualization and investigation. QW and XY: methodology. SH, WH, and YX: data curation, formal analysis, and software. HY, QW, and YX: validation and visualization. QT and HY: writing—original draft preparation. LW and ZJ: writing—review and editing, funding acquisition, project administration, and resources. All authors have read and agreed to the published version of the article.

## Funding

This study was funded by the National Key Research and Development Program of China (2018YFD0501101), China Agriculture Research System of MOF and MARA, the Science and Technology Program of Guangdong Academy of Agricultural Sciences (R2020PY-JG009), and Special fund for scientific innovation strategy-construction of high-level Academy of Agriculture Science (R2016YJ-YB2003, R2019PY-QF005, R2018QD-068).

## Conflict of Interest

The authors declare that the research was conducted in the absence of any commercial or financial relationships that could be construed as a potential conflict of interest.

## Publisher's Note

All claims expressed in this article are solely those of the authors and do not necessarily represent those of their affiliated organizations, or those of the publisher, the editors and the reviewers. Any product that may be evaluated in this article, or claim that may be made by its manufacturer, is not guaranteed or endorsed by the publisher.
